# Assessing practical laparoscopic training in certified Training Centers of the Gynecological Endoscopy Working Group (AGE) of the German Society of Gynecology and Obstetrics (DGGG)

**DOI:** 10.1007/s00404-019-05263-0

**Published:** 2019-08-21

**Authors:** Andreas Hackethal, Franz-Erich Solomayer, Uwe A. Ulrich, Sara Brucker, Bernd Bojahr, Bernd Holthaus, Stefan Rimbach

**Affiliations:** 1 Frauenklinik an der Elbe, Oberbaumbrücke 1, 20457 Hamburg, Germany; 2 Universitätsklinik Homburg/Saar, Kirrberger Str, 66424 Homburg, Germany; 3 Martin-Luther Krankenhaus, Caspar-Theyß-Straße 27-31, 14193 Berlin, Germany; 4 Universitäts-Frauenklinik, Calwerstrasse 7, 72076 Tübingen, Germany; 5 Klinik für MIC, Kurstraße 11, 14129 Berlin-Zehlendorf, Germany; 6 Krankenhaus St. Elisabeth gGmbH, Lindenstraße 3-7, 49401 Damme, Germany; 7 Krankenhaus Agatharied GmbH, Norbert-Kerkel-Platz, 83734 Hausham, Germany

**Keywords:** Laparoscopy training, Surgical education, Manual skills, Manual dexterity training

## Abstract

**Purpose:**

This study was performed to assess the practical laparoscopic training in Gynecological Endoscopy Working Group (AGE) certified Training Centers (TC) and evaluate the possible implementation for a manual dexterity skills-training within the Minimal Invasive Surgery (MIC) certification process.

**Material and methods:**

An online questionnaire was developed and the link provided for the heads of the AGE TC. The questionnaire comprised topics on TC organization, practical training performance and perspectives for future training and demographic data.

**Results:**

Response rate was 78.9% (15/19) of AGE TC. Grasping for the basic and suturing exercises for the advanced curricula, respectively, are thought to be of highest value (each 1.0 ± 0, on a scale from 1 = very valuable to 6 = not at all valuable). Most valuable parameter in assessing training was thought to be pressure/tension with 1.80 ± 1.08 The most valuable training capacity was considered for box training under supervision (1.27 ± 0.59) and feed-back box training with direct evaluation of various surgical skills (1.40 ± 0.63). Supervised box training was also thought to have the most positive influence on surgical performance (1.33 ± 0.49). The majority of respondents (86.7%) were qualified with the highest MIC certification and additional 66.7% were sub-specialized Gynecological Oncologists.

**Conclusion:**

The AGE certified TC offer a structured curriculum with emphasis on practical training. The results of this questionnaire and the additional respondents comments on value and future perspectives/changes of practical training support the concept and the implementation of a skills-training to the AGE MIC concept.

## Introduction

The Gynecological Endoscopy Working Group (AGE) strives for clinical excellence with a structured training and education curriculum in gynecological hysteroscopy and laparoscopy [1]. The AGE, founded in 1993, is part of the German Society of Gynecology and Obstetrics (DGGG) and represents the largest Workgroup of the German Society for Gynecology and Obstetrics (DGGG) with over 1800 members in 2019.

To standardize training and education as well as promote theoretical and clinical expertise, a graded certification module, Minimal Invasive Surgeon (MIC), was developed by the AGE board, discussed and optimized by the AGE council and accepted by the members during a general assembly in 2005 [1, 2]. Gynecological specialists and trainees as members of the AGE can apply for accreditation. During the last decade, there was a continuously increasing number of certified AGE members with 1144 certified members (MIC 1: 612, MIC II: 439, MIC III: 93) and 24 Training Centers in 2019. Requirements for application and certification are a defined number of completed hysteroscopic and laparoscopic procedures, conference and work-shop attendances and visits within other departments i.e. A full list of requirements for the different MIC certificates are listed in detail on the AGE homepage (www.ag-endoskopie.de). An overview of personal requirements for MIC and institutional requirements for Trainings Center certification are summarized in Tables 1 and 2.

**Table 1:** Summaries requirements for AGE MIC (Minimally Invasive Surgeon) I to III certification

	MIC I	MIC II	MIC III	MIC IIIa
Member of the AGE	*	*	*	*
Previous MIC certificate		MIC I	MIC I and MIC II	MIC I and MIC II
Certified Specialist Gynecology and Obstetrics		*	*	*
Basic curriculum	*			
Advanced curriculum		*		
Skills Training	*	*	*	*
Clinical visits			10 days	10 days
Attending 4 certified conferences		*	*	*
Number of performed laparoscopies	30 (Typ I)	400 (Typ II/III/IV) at least 20 Typ III/IV	800 (Typ II/III/IV) at least 80 Typ III/IV(°)	400 (Typ II/III/IV) at least 100 Typ III/IV(°)
From these: Number of assisted and supervised surgeries		up to 25%	up to 50%	up to 50%
Number of performed hysteroscopies	20 (diagnostic)	50 (operative)	60 (operative)	60 (operative)
From these: Number of assisted and supervised surgeries			up to 50%	up to 50%

^*^Requirement, °additional information available on AGE homepage

**Table 2:** The requirements for certified AGE Training Centers are summarized

AGE Training Center parameter	Requirements
Head	At least MIC II certified
Basic curriculum	Conduct at least one per year
Advanced curriculum	Conduct at least one per year
Operative Endoscopies	Perform at least 800 per year
Operative Laparoscopies (at least out of 4 categories)	Hysterectomy Myomectomy Lymphadenectomy Resektion of deep infiltrating endometriosis Suspension surgeries Organ sparing excisions Organ sparing ovarian cystectomies Organ sparing ectopic pregnancies
Operative Hysteroscopies (at least out of 3 categories)	Myomectomy Polyp resection Septum dissection Endometrium ablation Lysis of Synechia (III–IV°)
Visits	At least two participants

To further develop and add objective transparency to the MIC certification, the AGE board and members decided in 2016 to incorporate a scientifically based manual dexterity training or skills training to the already established individual certification requirements.

To better define and standardize specific box trainer or box trainer tasks and educational criteria for the skills training, this study aims to assess how the current practical laparoscopy training within certified AGE TC is performed and how to enquire on further perspectives of endoscopy training and skills training within the AGE TC.

## Material and methods

An online questionnaire with 29 questions in three parts was developed. The first part comprised nine questions on Training Center organization and their course volumes. The questionnaires second part assessed practical laparoscopic training parameters. Demographic data were collected within the last six questions of part three.

A google account was created for this study and the questionnaire was based on Google Drive platform. The questionnaire is attached in “Appendix 1”.

The heads of the AGE MIC Training Centers were invited by email to participate in this online survey between September and November 2016, with closing date 30th November 2016. At the end of October 2016 a reminder email was sent out. The answers were automatically saved on Google Drive and imported and analyzed with PSPP for iOS. Descriptive analysis was performed.

## Results

From 19 AGE certified Training Center (TC) in 2017, 15 (78.9%) completed questionnaires were available for evaluation. The respondent AGE TC were certified since a mean 4.86 ± 3.21 years. These 15 centers conduct 22 basic and 21 advanced curricula per year. Additional surgery courses were conducted by 67% (10/15) of these centers. These courses focus on special surgical technics, and are named and performed yearly: hysterectomy = 1, hysteroscopy = 1, endometriosis = 1, urogynecology = 4, myomectomy = 1, suturing skills = 1, radical hysterectomy = 1, cadaver workshop = 1, hospitation with observation of live surgery = 2.

Respondents see a focus on practical training compared to theoretical education as an important characteristic for the basic curricula (57.3% ± 17.9 vs. 40.7% ± 17.1) and the advanced curricula (58.7% ± 21.0 vs. 42.7% ± 19.8). For basic and advanced curricula, box training with different training models and Virtual Reality Simulators were more frequently used then box training with sensors to track instrument/target interactions (Table 3).

**Table 3:** An overview of what kind of practical laparoscopy trainer and training model is regularly used for basic and advanced curricula within the AGE Training Centers

	Basic curricula	Advanced curricula
Box trainer with improvised training models	9/15 (60%)	9/15 (60%)
Box trainer with standardized training models	9/15 (60%)	11/15 (73.3%)
Box trainer with commercially available standardized training models	9/15 (60%)	9/15 (60%)
Box trainer with sensors to track instrument/target interactions	1/15 (6.7%)	1/15 (6.7%)
Virtual Reality Simulator	6/15 (40%)	7/15 (46.7%)

While grasping exercises were thought to be most valuable training tools for the basic curricula, needle movement and suture exercises were thought to be most valuable in advanced curricula (Table 4). Additionally, respondents valued suture training and techniques combined with standardization of suturing as highly appreciated tasks for the basic and the advanced curriculum (Table 4). For the Basic Curriculum suggested additional tasks were: 1.Use of 0° and 30° scopes and coordination (3×), 2. Suture techniques (3×), 3. Dissection techniques (1×) and for the Advanced Curriculum: 1. Dissection with demonstration of anatomical structures (1×), 2. Simulation of surgical procedures (2×), 3. Complication management (×2), 4. Standardized suture techniques (4×) and 5. Practical training with objective measurement of skills (1×).

**Table 4:** Summarizes answers on value of box trainer tasks, participants evaluation, box trainer features and task models from (1 = very valuable, 6 = not at all valuable)

Question	Parameter	(1 = very valuable/important to 6 = not at all valuable/important)
Box trainer tasks to be valuable for training in basic curriculum?	Grasping exercise	1.00 ± 0
	Cutting exercise	1.47 ± 1.30
	Needle movement exercise	1.47 ± 0.74
	Suture exercise	2.27 ± 1.28
Box trainer tasks to be valuable for training in advanced curriculum?	Grasping exercise	2.53 ± 1.68
	Cutting exercise	2.67 ± 1.80
	Needle movement exercise	1.13 ± 0.35
	Suture exercise	1.00 ± 0
Parameter to be valuable for evaluation of participants box training?	Time (to finish a task)	1.93 ± 0.96
	Instrument movement (Effectiveness in cm to finish a task)	2.00 ± 0.85
	Errors (i.e dropping object)	2.27 ± 1.10
	Pressure/tension (i.e for evaluation of tissue handling)	1.80 ± 1.08
Which characteristics and features are important to be combined with the ideal box trainer?	Easy to set up and flexible	1.27 ± 0.46
	To have adjustable height	2.33 ± 1.11
	Enable flexible trocar positioning	1.87 ± 1.41
	Assess and save training data to enable learning curve	2.27 ± 0.70
	Usable with plastic and organic models	2.73 ± 1.10
	Combinable device, allowing to track instrument movement and coordination	2.20 ± 0.86
	Device allowing to assess pressure and tension at task model	2.27 ± 1.03
	Ability to choose instrument	2.07 ± 1.39
Which features of task models for the box trainer are of important value?	Easy to purchase	1.73 ± 0.70
	Training of relevant procedures (i.e Suturing)	1.33 ± 0.62
	Cost effective and reusable	1.53 ± 0.83
	Ideally close to reality (i.e simulation of bleedings)	2.20 ± 1.32
	Structured and with instructions	1.53 ± 0.74
	Comparable to other courses	1.80 ± 0.94
	Easy to clean	1.60 ± 0.74

The respondents thought, there is no parameter clearly superior for evaluating participants during box training, however, assessed pressure/tension for the evaluation of tissue handling was thought to be most valuable (Table 4). Additionally, 6/15 respondents suggested further parameter to be of value. These comprised measuring blood loss in virtual reality environment, repetition of tasks and skills, tissue dissection and tissue handling, strategy in solving tasks, following instructions. Most important characteristics and features of box trainer are thought to comprise easiness and flexibility for set up and allowing for individual trocar positioning. The training models and tasks should be train relevant procedures in a structured way and be cost-effective and reusable (Table 4).

The most valuable training capacity (1 = very valuable, 6 = not at all valuable) was considered for box training under supervision (1.27 ± 0.59) and feedback box training with direct evaluation of various surgical skills (1.40 ± 0.63) (Table 5). Supervised box training was also thought to have the best positive influence on surgical performance (1.33 ± 0.49) (Table 5).

**Table 5:** Summarizes estimated training capacity, ability to evaluate the participant and positive influence on surgical performance with different training models (1 = very valuable, 6 = not at all valuable)

Training model	Training capacity	Ability to evaluate the participant	Positive influence on surgical performance
Box training	2.13 ± 0.99	2.53 ± 0.92	2.13 ± 0.83
Box training under supervision	1.27 ± 0.59	1.87 ± 0.74	1.33 ± 0.49
Curricula based box training (standardized tasks)	1.67 ± 0.72	1.87 ± 0.74	1.47 ± 0.52
Feed-back box training with direct evaluation of various surgical skills (instrument movement, tissue handling, errors, time)	1.40 ± 0.63	1.60 ± 0.63	–
Virtual Reality Simulation training with feedback	1.93 ± 0.88	1.93 ± 0.88	1.87 ± 0.92

Further considerations on value of training for the AGE courses were given from 67% (10/15) of participants. These comprise:

1. Stepwise training with animal lab and cadaver lab for experts.
2. Maximum of two participants per box trainer only.
3. Defined goal for practical training.
4. Standardization of practical laparoscopy training in between centers.
5. Courses should always comprise practical skills-training.
6. Complications management.

### Demographic data

Respondents mean age was 47.5 years (SD 6.3), whilst 46.7% (7/15) were clinic directors, with the remaining being senior consultants 26.7% (4/15) and private practitioners 26.7% (4/15). Ten respondents (66.7%) were additionally sub specialized Gynaecological Oncologists; the majority were qualified as MICIII surgeons [86.7% (13/15)]. The total number of responsible held laparoscopic training courses differed individually between less than 20 (6/15, 40%) to more than 80 courses (2/15, 13.3%), whilst the remaining 7/15 56.7% held between 20 and 80 courses.

Ten respondents addressed answers to most important changes in laparoscopic surgical education, these were thought to be 1. standardization (4×), 2. more and available Box training (3×), measuring training (1×), inclusion of MIC training and certification into specialization training.

## Discussion

The institutionally performed endoscopic training within the certified Gynecological Endoscopy Working Group (AGE) Training Centers (TC) in Germany was assessed and evaluated in this study. With fifteen responding AGE TC and over 55 structured endoscopic basic and advanced training courses per year, the AGE TC are dedicated to surgical training with comprehensive experience especially in the field of endoscopic surgery. Notably, two third of TC conduct additional laparoscopy courses, apart from the AGE required curricula, which are mostly disease or organ-related procedural courses (urogynecology, hysterectomy courses i.e.).

Practical training plays an important role in the AGE TC training curricula with approximately 60% of course time. The importance of practical training is supported by scientific results showing box training on models and Virtual Reality Training being able to distinguish between novices and experts and directly translate to improved theatre performance and patient outcome [3–7].

Additionally, valuable training tasks are thought to include mainly coordination training within the basic and suture training within the advanced endoscopy courses.

The AGE TC mostly use box training and virtual reality (VR) training for the practical education. They favor standardized, easy to assemble and flexible box trainer with cost effective and reusable models simulating relevant surgical procedures. The variety of examined and published box trainer tasks ranges from raw swaps for suturing to costly and complex installable pulsating organ perfusion (POP) models [8, 9]. Furthermore, VR Trainer allow for standardized training in an abstract or near realistic environment [10]. Whereas assessment of skills during box training is mostly subjective by the supervisor or has limited validity, such as time measurement for task completion, more advanced box trainer and VR Trainer assess various parameter (i.e. distance of instrument movement, force/traction and errors).

The inclusion of VR into AGE TC and endoscopy courses is mainly a result of the AGE purchasing a VR Hysteroscopy Trainer in 2010, which can be requested by TC for educational courses. Therefore, experience with VR-trainers, the benefits of objective skills-assessment and direct feedback has gained an important role within the TC. Even though a recent meta-analysis found better laparoscopy performance and time scores in VR training compared to box-training, all other evaluated parameter, regardless of the student's level of experience or type of activity, were equivalent [11].

In our study, AGE TC favor box trainer with sensors to objectively measure instrument movement, tissue handling, errors and time for the ability to evaluate the trainee. Therefore it is likely that TC add more advanced box trainer with objective skill assessment to their curriculum. Additionally, TC estimated all examined parameters to have a valuable role in skills assessment with pressure/tension being the most valuable and errors being the least valuable parameter in this study.

Technically these advanced objective assessments can be done by sensor equipped box trainer, such as the ForceSense system (Medishield) or VR Trainer, such as the LapSim (Surgical Science) [12, 13]. However, the AGE TC clearly favor the sensor equipped box trainer over VR trainer. The similar ability to objectively assess trainees and receive instant score results after practice as well at the systems price difference may be an important reason. More than that, VR Trainer are technically more susceptible and the maintenance is more complex compared to box trainer.

The AGE graded sub-certification and the MIC certificate is unique and has become an important qualification tool within the German speaking gynecological field. Other gynecological societies as the International Society for Gynecological Endoscopy (ISGE), the Australian Gynaecological Endoscopy Society (AGES), the British Society for Gynaecological Endoscopy (BSGE) or American Association of Gynecological Laparoscopy (AAGL), to name a few, organize sub-specialized endoscopy courses with main focus on practical training as well, but without a pre-defined curriculum or skills-assessments. Only the European Society of Gynecological Endoscopy (ESGE) promotes a complex educational concept, allowing for graded accreditation [14].

The group of respondents are highly experienced and specialized practitioners with 13 out of 15 being MIC III certified and 67% being sub-specialized in gynecologic oncology. The results of this questionnaire and the additional respondents comments on value and future perspectives/changes of practical training, mainly expecting more standardization and box training with objective assessment tools, are the reason for the AGE to further develop the concept and the implementation of a skills-training to the AGE MIC concept.
